# Prediction of pathogenic single amino acid substitutions using molecular fragment descriptors

**DOI:** 10.1093/bioinformatics/btad484

**Published:** 2023-08-03

**Authors:** Anton Zadorozhny, Anton Smirnov, Dmitry Filimonov, Alexey Lagunin

**Affiliations:** Department of Bioinformatics, Pirogov Russian National Research Medical University, Moscow 117513, Russia; Department of Bioinformatics, Pirogov Russian National Research Medical University, Moscow 117513, Russia; Department of Bioinformatics, Institute of Biomedical Chemistry, Moscow 119992, Russia; Department of Bioinformatics, Pirogov Russian National Research Medical University, Moscow 117513, Russia; Department of Bioinformatics, Institute of Biomedical Chemistry, Moscow 119992, Russia

## Abstract

**Motivation:**

Next Generation Sequencing technologies make it possible to detect rare genetic variants in individual patients. Currently, more than a dozen software and web services have been created to predict the pathogenicity of variants related with changing of amino acid residues. Despite considerable efforts in this area, at the moment there is no ideal method to classify pathogenic and harmless variants, and the assessment of the pathogenicity is often contradictory. In this article, we propose to use peptides structural formulas of proteins as an amino acid residues substitutions description, rather than a single-letter code. This allowed us to investigate the effectiveness of chemoinformatics approach to assess the pathogenicity of variants associated with amino acid substitutions.

**Results:**

The structure-activity relationships analysis relying on protein-specific data and atom centric substructural multilevel neighborhoods of atoms (MNA) descriptors of molecular fragments appeared to be suitable for predicting the pathogenic effect of single amino acid variants. MNA-based Naïve Bayes classifier algorithm, ClinVar and humsavar data were used for the creation of structure-activity relationships models for 10 proteins. The performance of the models was compared with 11 different predicting tools: 8 individual (SIFT 4G, Polyphen2 HDIV, MutationAssessor, PROVEAN, FATHMM, MVP, LIST-S2, MutPred) and 3 consensus (M-CAP, MetaSVM, MetaLR). The accuracy of MNA-based method varies for the proteins (AUC: 0.631–0.993; MCC: 0.191–0.891). It was similar for both the results of comparisons with the other individual predictors and third-party protein-specific predictors. For several proteins (BRCA1, BRCA2, COL1A2, and RYR1), the performance of the MNA-based method was outstanding, capable of capturing the pathogenic effect of structural changes in amino acid substitutions.

**Availability and implementation:**

The datasets are available as supplemental data at Bioinformatics online. A python script to convert amino acid and nucleotide sequences from single-letter codes to SD files is available at https://github.com/SmirnygaTotoshka/SequenceToSDF. The authors provide trial licenses for MultiPASS software to interested readers upon request.

## Graphical Abstract

**Graphical Abstract:**
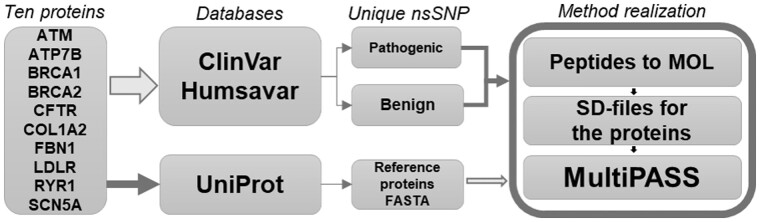

## 1 Introduction

In 2021, the global market for the next generation sequencing (NGS) was estimated at $6.37 billion (https://www.precedenceresearch.com/next-generation-sequencing-market), corresponding to approximately 7 million human genome-wide sequences. NGS allows to detect any genetic variant in an individual patient, serving as a bridge between contemporary and precision medicine. Those variants include single nucleotide polymorphisms (SNPs, also known as single nucleotide variants, SNVs), insertion and deletion, copy number variation and chromosomal rearrangement events. Since SNPs make up the majority variants [e.g. dbSNP contains over 1 billion records,  (Sherry *et al.*, 2001, https://www.ncbi.nlm.nih.gov/snp)], the greatest interest present nonsynonymous variants leading to a change in the amino acid (a.a.) sequence. Amino acids substitutions (single amino acid variants, SAVs), that lead to the disarrangement of normal functions of proteins, are deleterious, otherwise they are benign. Molecular consequences for some SNPs have annotations in specific databases (such as UniProt, HGMD, ClinVar, etc.). But most of missense SNPs related with SAVs have no experimental or/and clinical reports with annotations describing their pathogenicity. Thereby, predicting the SNP effect using computational tools is a common practice. Such tools may differ in mathematical algorithms, the used characteristics of amino acids/nucleotide sequences, and in the scoring system. Two common strategies in developing pathogenicity predictors are the combination of properties driven by evolutionary/physicochemical consideration (i.e. individual tools) and aggregation of pre-existing pathogenicity predictors scores to produce new predictions (metapredictors) (Özkan *et al.* 2021). Individual tools are trained on large annotated data, various features may be in input vector including evolutionary (Carter *et al.* 2013, Wang *et al.* 2020, Won *et al.*, 2021), conservation information (Li *et al.* 2009, Vaser *et al*. 2016, Malhis *et al.* 2020), and functional estimates (Capriotti *et al.* 2013, Niroula *et al.* 2015, Pejaver *et al.* 2020). In addition, diverse methods utilize properties of amino acids (Adzhubei *et al.* 2013, Reva *et al.* 2011, Qi *et al.* 2021) and proteins sequence context (Calabrese *et al.* 2009, Choi *et al.* 2012, López-Ferrando et al. 2017, Ancien *et al.* 2018). On the other hand, metapredictors (Jagadeesh *et al.* 2016, Kim *et al.* 2017) emphasize the adaptation of classification algorithms, such as Random Forests or Neural Networks, rather than leveraging unique features.

Despite the abundance of such software, the prediction of SAVs function impact remains a challenging and unsolved problem. The recent comparing of the methods (López-Ferrando et al. 2017) on selected number of genes has shown an inferior prediction accuracy compared to the average score obtained in the global test. The authors conclude that there exists the necessity to evolve specific predictors for protein families exhibiting nonstandard behavior. The decreased accuracy may be explained using heterogenic data in training models. Moreover, because all selected genes are associated with distinct diseases, the tools may be incompletely used in making clinical decisions. Protein-specific (PS) pathogenicity prediction strategy supposed data of specific gene/protein can complement current tools. Recent studies have shown the performance competitiveness of specific tools with nonspecific predictors (Torkamani and Schork 2007, Crockett *et al.* 2012, Riera *et al.* 2016), with a tendency to outperform them (Riera *et al.* 2016).

In chemoinformatics, such an approach is known as structure-activity relationship analysis (SAR), and it is successfully implemented in computer-aided drug design. Basically, SAR analysis describes the determination between a chemical structure represented in machine-readable formats and a biological activity of compounds (Tong *et al.* 2003, Guha 2013, Muratov *et al.* 2020). Besides, SAR models are usually built for a specific target without considering it directly. Since many targets and biological activities are sparsely studied, SARs are often applied to small or unbalanced datasets (Idakwo *et al.* 2020, Sakai *et al.* 2021). The main concept in this work is to consider amino acid sequences of peptides with centered SAV as structural formulas. Such descriptions may provide additional information for SAVs pathogenicity effect. Thus, we propose an approach for predicting the pathogenicity impact of SAVs in clinically relevant proteins consisting of PS classifiers trained on structural SAVs with the nearest amino acid neighbors representation.

## 2 Materials and methods

### 2.1 Datasets and data preparation

**Figure 1.**
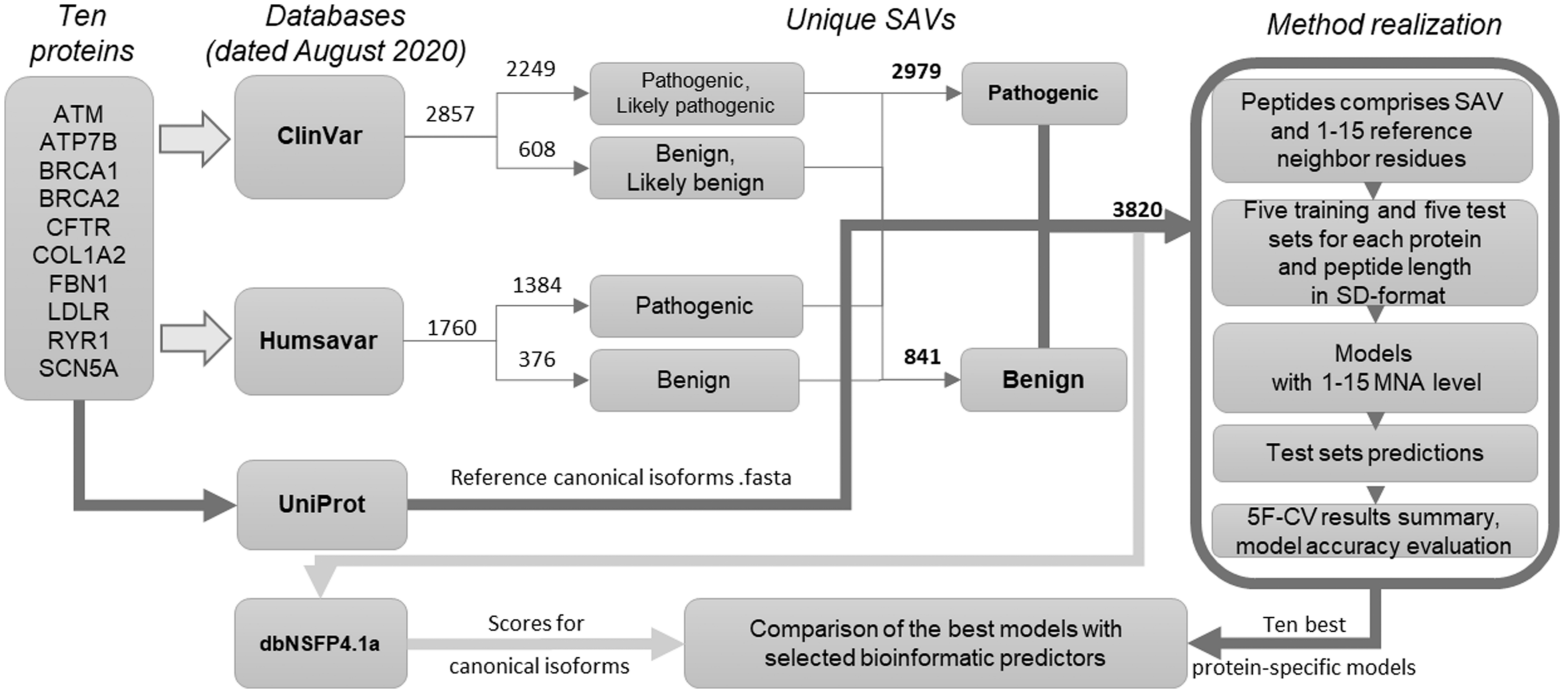
The scheme of the study. The studied proteins are represented as the gene symbols. Annotated single amino acid variants (SAVs) were taken from ClinVar and Humsavar databases. The numbers under the arrows are the total number of SAVs. UniProt was used as a source of protein sequences. Based on protein sequences, peptides with different sizes were created (depending on the number of amino acids close to the central SAV). The models were trained only on the peptides with protein-related substitutions represented in structured-data (SD) format. Test sets predictions were made on corresponding training sets during the 5-fold cross validation procedure (5F-CV). For comparison, pathogenicity scores were obtained from the academic version of dbNSFP4.1 for 11 bioinformatic predictors. MNA, Multilevel Neighborhoods of Atoms descriptors.

The schematic diagram of the study is displayed in Fig. 1. The research object consists of 10 disease associated proteins [selected from the publication of López-Ferrando co-authors (López-Ferrando et al. 2017)] and their known missense variants from ClinVar (Landrum *et al.* 2018) and humsavar (http://www.uniprot.org/docs/humsavar) databases dated August 2020. According to the ACMG Guidelines (Richards *et al.* 2015) classification used in ClinVar, “pathogenic” or “likely pathogenic” variants were indicated as “pathogenic” (1), likewise “benign” or “likely benign” composed as “benign” (0) class. All 2857 ClinVar selected records had minimum “one star” review status. The data from humsavar (1760 SAVs) was applied as previously classified (humsavar.txt file, released 26 February 2020). Subsequently, the overlap between two datasets was executed and conflicts in variants interpretation were resolved (depending on the last valid record date), the resulting 3820 SAVs, 841 benign and 2979 pathogenic, were recognized. Twenty records had conflicts in clinical interpretation, for most of them annotations from ClinVar were chosen. Only three variants had more recent entries in the humsavar database that were selected for the study. The general sizes of the datasets with the number of pathogenic and benign SAVs for each protein are represented in Table 1. The proteins had both pathogenic and benign SAVs, with minimum of 125 in a dataset, which made it possible to create more stable classification models. Since some chosen proteins have several known isoforms, the reference canonical sequences pursuant to SwissProt (The UniProt Consortium, 2021) were used.

**Table 1.** Dataset structure and coverage regarding dbNSFP4.1a.

Gene	PL^a^	Dataset^b^	Pathogenic	Benign	dbNSFP^c^	Coverage (%)^d^	
ATM	3056	129	54	75	125	96.9	
ATP7B	1465	249	215	34	249	100	
BRCA1	1863	321	126	195	318	99.0	
BRCA2	3418	329	79	250	328	99.6	
CFTR	1480	239	209	30	245	100	
COL1A2	1366	289	258	31	287	99.3	
FBN1	2871	1013	984	29	1011	99.8	
LDLR	860	704	618	86	700	99.4	
RYR1	5038	275	228	47	273	99.2	
SCN5A	2016	272	208	64	270	99.2	
Summary		3820	2979	841	3800	99.4	

a PL, protein length in amino acids.

b Number of single amino acid substitutions in the dataset.

c Number of the substitutions from the dataset found in dbNSFP4.1a.

d Coverage = (dbNSFP/Dataset) * 100.

**Figure 2.**
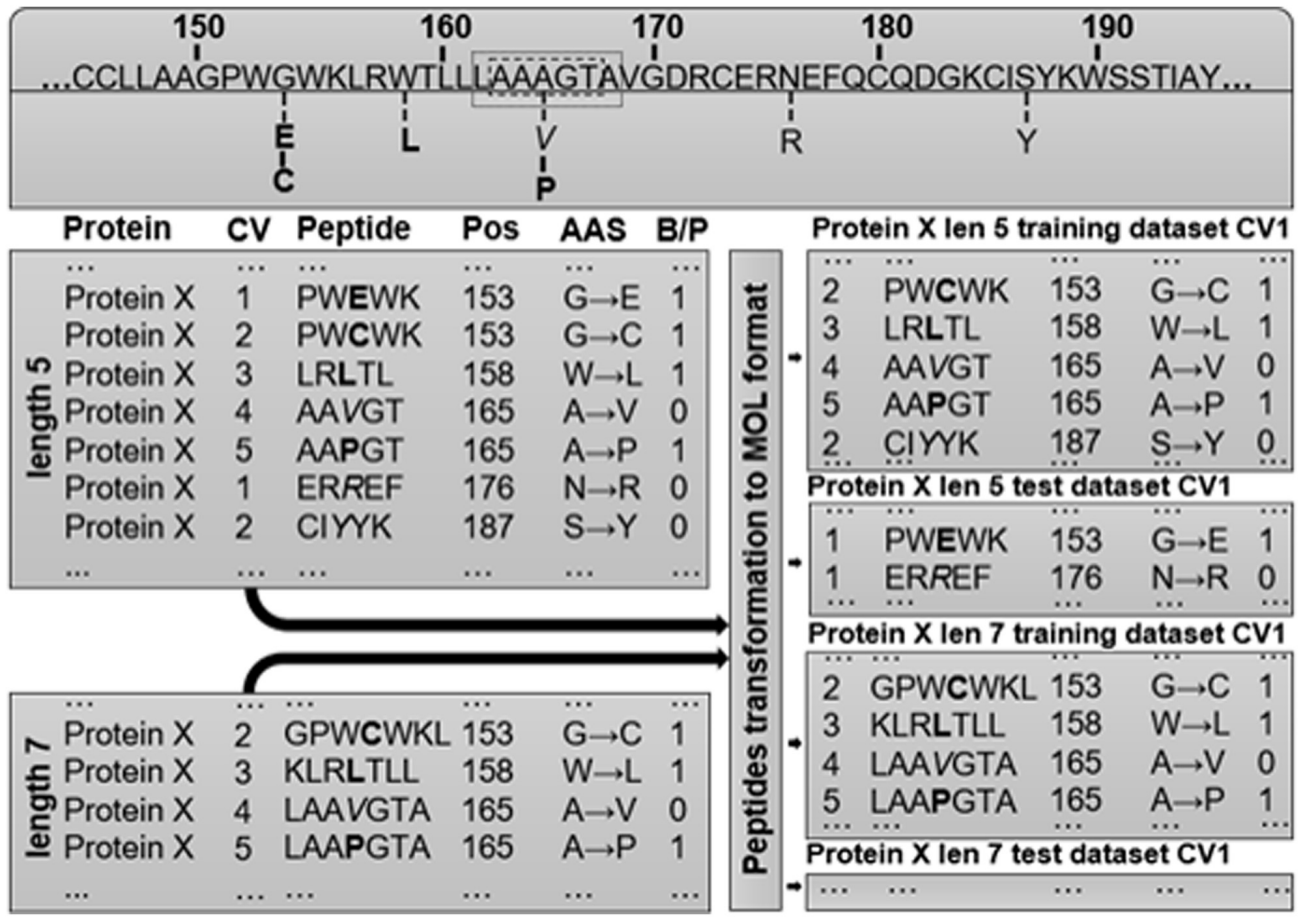
Illustration of obtaining datasets. A fragment of a hypothetical protein X, with highlighted SAVs located at positions from 150 to 190. Pathogenic (P, 1) are bold, benign (B, 0) are italics. Training and test datasets are derived from protein X and contain peptides of a certain length. SD-files record includes the peptide structure in the MOL format as well as the SAV effect label and accompanying information.

The method concept of dataset creation for training and validation of SAR models is illustrated at Fig. 2. Firstly, every SAV was mapped to corresponding sequences of related protein and peptides with the length from 3 to 31 a.a. residues were obtained, respectively. The fragments had SAV in the center of peptides and 2–15 neighbors as natural amino acids. SAVs located at the edges of the protein shift from the fragment center. In summary, we had 15-peptide lengths for 10 selected proteins that were compiled to 150 structure-data files (SDFs). An SDF contains structural formulas of definite protein fragments described in MOL V3000 format, the SAV position, the coding protein gene name, and the effect of missense variant labels. Next, SDFs were divided into five training and five test datasets to make 5-fold cross-validation (5F-CV). Datasets generations, including fragments translation to structural formulas, were made with the original Python script. In the end, test sets contained unique peptides with the fixed length from distinct positions of the protein, similarly, training sets incorporated the remaining fragments. The division was taken into account sorting by the position label, resulting at least 100 records for training and 25 for test datasets, respectively. The datasets are avaible as CSV files in Supplementary materials.

### 2.2 PASS software and Multilevel Neighborhoods of Atoms (MNA) descriptors

**Figure 3.**
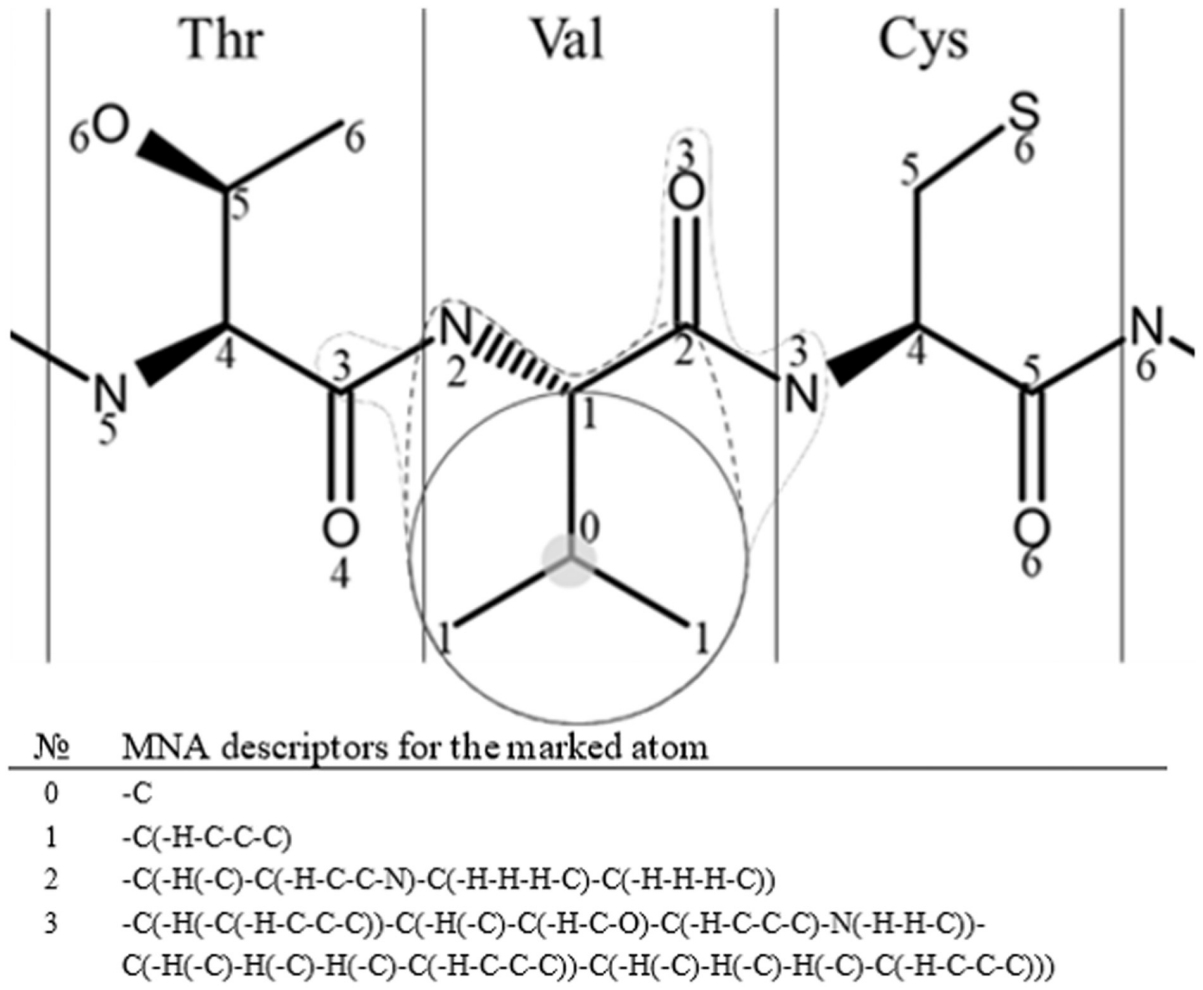
MNA descriptors are calculated for the C-atom of the valine (Val) residue in the polypeptide chain fragment. The numbers display the most distant atoms included in the descriptor of corresponding level. The selected MNA level is used in a model, sets of such descriptors are generated for the each of atoms. That allows to describe the peptide structure entirely and unequivocally.

To create classification models predicting the effect of SAVs, a special version of PASS (Prediction of Activity Spectra for Substances) software (MultiPASS version) was used. The PASS software was successfully implemented into several web applications to predict the biological activity of compounds based on their structural formula (Lagunin *et al.* 2000, Lagunin *et al.* 2013, Rudik *et al.* 2015, Lagunin *et al.* 2018). MultiPASS version was made specifically for amino acid and nucleotide sequences structure analysis. MultiPASS employs a uniform set of atom-centric substructural MNA descriptors for the representation of peptide structures and a modified Naïve Bayes classifier to model structure-activity relationships (Poroikov et al. 2000, Filimonov et al. 2014). Earlier this approach was successfully used to predict phosphorylation sites of proteins (Karasev *et al.* 2017) and epitope/MHC specificity for CDR3 TCR sequences (Smirnov *et al.* 2023). MNA descriptors are based on the molecular structure representation that includes hydrogen atoms in accordance with the valences and partial charges of atoms and does not specify bond types (Filimonov et al. 2014). MNA descriptors for each atom of the molecule are computed recursively as follows: the 0th level is the mark A of the atom itself (*D*_0_(*A*)= (−)*A*), where “−” is a mark added to nonring atoms, and any next level descriptor is the linear substructure notation *D_n_*(*A*) = *D_n_*_–1_(*A*)(*D_n_*_–1_(*B*_1_)*D_n_*_–1_(*B*_2_)…*D_n_*_–1_(*B_k_*)), *D_n_*_−1_(*B_i_*) is the (*n *−* *1)-level MNA descriptor for atom A’s *i*-th immediate neighbor *B_i_*. According to the notation, descriptors include various distanced atom neighbors, and higher levels include previous ones (Fig. 3). Since the original PASS deals with small molecules, it uses 1–2 levels of MNA descriptors. In turn, the main feature of MultiPASS is the calculation of a higher-level of MNA descriptors (up to 15), which provides a possibility to describe the structure of macromolecules such as peptides.

For each predictable “activity”, the PASS algorithm calculates two probabilities (Pa and Pi) for the studied compound. Pa (probability “to be active”) and Pi (probability “to be inactive”) estimates the belonging of the predicted compound to classes of active and inactive compounds, respectively (Filimonov et al. 2014). We used (Pa-Pi) > 0 as a final score for the pathogenic class in the predictions. Thus, models were built on diverse peptides lengths and MNA-level combinations.

### 2.3 Approach estimation

All protein classifiers were trained according to the 5F-CV procedure using from 1th to 15th MNA levels, which implies each one-fifth part of the dataset was used as an independent test set and each four-fifth part was used as a training set (Fig. 2). The predictions on test sets were summarized to compare the quality of models as the area under the receiver operating characteristic curve (AUC) and Mathew’s correlation coefficient (MCC) measures, as well as sensitivity, specificity, and balanced accuracy metrics. where MCC is the Matthew Correlation Coefficient MCC, TP is the true positive, TN is the true negative, FP is the false positive, and FN is the false negative.


$$\mathrm{MCC}=\;\frac{\mathrm{TP}\times \mathrm{TN}-\mathrm{FP}\times \mathrm{FN}}{\sqrt{\left(\mathrm{TP}+\mathrm{FP})(\mathrm{TP}+\mathrm{FN})(\mathrm{TN}+\mathrm{FP})(\mathrm{TN}+\mathrm{FN}\right)}}$$

To make a comparative test between the MNA-based approach and several existed bioinformatic predictors, the prediction scores were taken from dbNSFP4.1a (Liu *et al.* 2020). The academic version of dbNSFP4.1 contains transcript-specific functional predictions scores and interpretations for human nonsynonymous and splice-site SNPs (Liu *et al.* 2020) made by the computational tools. We chose the scores belonging to protein canonical isoforms. The total number of SAVs is represented in Table 1. The cut-off points of appropriate methods were used for AUC and MCC calculation. The assessment metrics were gained with the original Python script and Scikit-learn library (Pedregosa *et al.* 2011).

## 3 Results

### 3.1 MNA-based method performance

**Figure 4.**
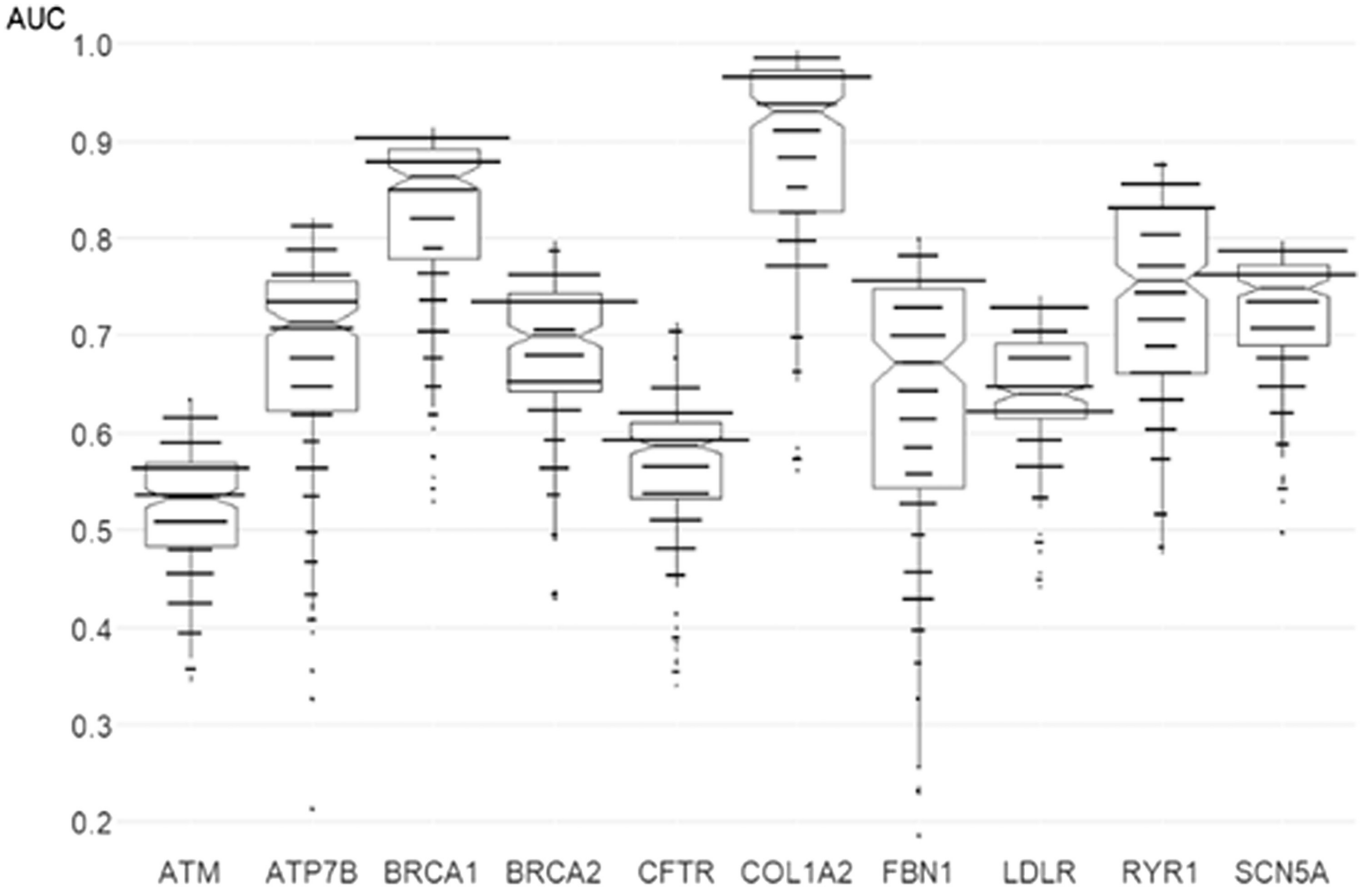
Overall performance of protein-specific models based on different peptide length and MNA level in 5-fold cross-validation. The boxplots represent the varying range of accuracy for the models; long tails are usually caused by a lower informativeness of 3–5 peptide fragments and 1–3 MNA level descriptors. The dots represent the number of models with a particular AUC value, a total of 225 per protein.

Overall, 150 datasets and 11 thousand SAR models were built. We studied how the length of peptides and the level of MNA descriptors influenced the classification models accuracy during 5F-CV procedure. It appeared that the models differ over a wide range of AUC and MCC values (Fig. 4, Supplementary Fig. S1). Variants effects in some proteins (e.g. ATM and CFTR) are poorly predicted by the structural description, as most of those models have the AUC parameter below 0.7. Nevertheless, several individual protein models have a better predictive power. A large range of AUC scores for FBN1 was given for SAR models created on different peptide length and levels of MNA descriptors. It may be caused by the predominance of pathogenic over neutral substitutions in the dataset (Table 1). During the study, we could not identify certain peptide length or MNA level parameters for the method which would lead to best SAR models on all proteins (Supplementary Fig. S1). Instead, for each protein we selected the combinations of the peptide length and levels of MNA descriptors leading to the highest values of AUC. The list of proteins associated with diseases and best models’ evaluation characteristics is given in Table 2. Self-recognition and the reference peptide tests were also made (Supplementary Table S1). The models perform with an approximately 100% accuracy in the self-recognition test. In the reference peptide test, the benign labels for unmutated peptides (randomly generated from the reference sequence of the protein) were predicted with a similar accuracy tendency as for 5F-CV results. We studied the correlation between AUC values and the size of training sets, proportions of pathogenic and benign variants. There appeared to be no correlation between AUC values of models and the number of variants or their proportions in the training sets. (Supplementary Fig. S2). According to superior mean values of AUC and MCC scores, we chose models with optimal parameters for every protein, mainly 9–13 levels for MNA descriptors and 15–31 for peptide length. In case of close scores, we chose classifiers built on lower parameters (Table 2, the parameters are put in Table 3).

**Table 2.** Ten investigated proteins descriptions with the highest performance metrics for MNA-based predictions during 5F-CV procedure.^a^

Gene	UniProtKB	Protein	Disease	Sen.	Spec.	BA.	MCC	5F-CV	LOO-CV
ATM	Q13315	Serine-protein kinase ATM	Hereditary CPR	0.667	0.547	0.607	0.211	0.631	0.631
ATP7B	P35670	Copper-transporting ATPase 2	Wilson disease	0.833	0.765	0.799	0.474	0.808	0.815
BRCA1	P38398	Breast cancer type 1 susceptibility protein	Breast-ovarian cancer, familial 1	0.794	0.923	0.858	0.730	0.907	0.900
BRCA2	P51587	Breast cancer type 2 susceptibility protein	Breast-ovarian cancer, familial 2	0.747	0.728	0.737	0.417	0.797	0.780
CFTR	P13569	Cystic fibrosis TCR	Cystic fibrosis	0.684	0.633	0.659	0.220	0.695	0.712
COL1A2	P08123	Collagen alpha-2(I) chain	Osteogenesis Imperfecta	0.973	1.000	0.986	0.891	0.993	0.992
FBN1	P35555	Fibrillin-1	Marfan syndrome	0.768	0.724	0.746	0.191	0.795	0.789
LDLR	P01130	Low-density lipoprotein receptor	Familial hypercholesterolemia	0.728	0.709	0.719	0.306	0.743	0.730
RYR1	P21817	Ryanodine receptor 1	Central core disease	0.833	0.830	0.832	0.556	0.872	0.875
SCN5A	Q14524	Sodium channel protein type 5 subunit α	Brugada syndrome	0.740	0.703	0.722	0.391	0.765	0.724

a AUC, area under the receiver operating characteristic curve; 5F-CV, AUC obtained in five cross-validation procedure; LOO-CV, AUC obtained in leave-one-out validation procedure; Sen., sensitivity; Spec., Specificity; BA, balanced accuracy; MCC, Matthew correlation coefficient; ATM, ataxia-telangiectasia mutated; CPR, cancer-predisposing syndrome; TCR, transmembrane conductance regulator.

**Table 3.** The comparative performance of the predictors on selected proteins.^a^

	MNA-based predictions					SIFT 4G				PROVEAN				PolyPhen 2 HDIV				MutationAssessor				FATHMM			
Gene (Protein)	Len	MNA	AUC	MCC	%		AUC	MCC	%		AUC	MCC	%		AUC	MCC	%		AUC	MCC	%		AUC	MCC	%
ATM	21	14	0.631	0.211	100		0.840	0.544	96		**0.880**	**0.601**	**96**		0.853	0.496	96		0.786	0.448	96		0.649	0.213	96
ATP7B	23	12	0.808	0.474	100		0.873	0.451	100		**0.883**	**0.643**	**100**		0.864	0.607	100		0.822	0.345	100		0.702	0.160	100
BRCA1	31	13	**0.907**	**0.730**	**100**		0.885	0.478	99		0.567		99		0.743	0.450	99		0.753	0.380	96		0.691	0.139	99
BRCA2	15	12	**0.797**	**0.417**	**100**		0.748	0.368	99		0.620		99				0				0		0.711	0.361	99
CFTR	15	10	0.695	0.220	100		0.610	0.239	100		0.733	0.246	100		0.678	0.211	100		0.738	0.203	100		0.489	0	100
COL1A2	9	9	**0.993**	**0.891**	**100**		0.925	0.649	99		0.964	0.807	99		0.920	0.641	99		0.982	0.742	99		0.967	0.309	99
FBN1	11	13	0.795	0.191	100		0.821	0.314	99		0.899	0.364	99				0				0		0.811	0.096	99
LDLR	15	13	0.743	0.306	100		0.848	0.502	99		**0.867**	**0.604**	**99**		0.840	0.519	99		0.860	0.495	99		0.794	0	99
RYR1	21	11	**0.872**	**0.556**	**100**				0		0.775	0.420	98		0.767	0.463	98		0.811	0.398	98		0.689	0.210	98
SCN5A	15	9	0.765	0.391	100		0.809	0.387	99		0.813	0.349	96		0.787	0.337	98		0.785	0.384	99		0.815	0	99
**Mean**			0.801	0.439	100		0.818	0.437	89		0.800	0.376	99		0.810	0.466	79		0.817	0.424	79		0.734	0.149	99

a Len, peptide length parameter of the model; MNA, MNA level parameter of the model; AUC, area under the ROC curve; MCC, Matthew correlation coefficient; %, percentage of the tools’ scores presented in dbNSFP4.1a. The best protein performance for the individual methods are in bold.

We matched the MNA-based scores with the original datasets and related them to the primary structure with applied domains from Pfam database (Mistry *et al.* 2021), based on the calculation of SAV frequency in 100 a.a. sliding window (Supplementary Fig. S14). For each position we calculated the number of pathogenic or benign variants in the window, the obtained values were divided by 100. Supplementary Fig. S3 shows that for some proteins the distribution of known pathogenic and benign SAVs varies along positions. For example, BRCA1 sequence has many pathogenic variants on the edges, and most benign variants are located in other parts of the protein. For proteins with such a controversial distribution of pathogenic and benign variants, we got classification models with the highest value of accuracy. In addition, AUC values of the models built on wholly related datasets (Tables 3 and 4) were calculated by the leave-one-out cross-validation (LOO-CV) and the 20-fold validation (Supplementary Table S1, Supplementary Fig. S2C) procedure during the training. Analyzing AUC 5F-CV and AUC LOO-CV values offered no significant differences between them (Mann-Whitney test, *P* > 0.05). All of the statements above suggest the PS models are robust.

**Table 4.** The comparative performance of the predictors on selected proteins.^a^

	MVP			LIST-S2			MutPred			M-CAP			MetaSVM			MetaLR		
Gene (Protein)	AUC	MCC	%	AUC	MCC	%	AUC	MCC	%	AUC	MCC	%	AUC	MCC	%	AUC	MCC	%
ATM	0.834	0.261	91	0.719	0.300	96	0.951	0.801	58	0.835	0.329	79	0.718	0.285	99	0.766	0.485	96
ATP7B	0.873	0.359	97	0.856	0.476	100	0.985	0.835	82	0.888	0	95	0.901	0.481	96	0.891	0.485	100
BRCA1	0.846	0.430	96	0.819	0.423	99	0.862	0.502	43	0.897	0.139	94	0.847	0.595	100	0.808	0.427	99
BRCA2	0.781	0.187	97			0	0.802	0.474	63	0.713	0.14	92	0.676	0.561	99	0.742	0.344	99
CFTR	0.633	0	99	0.631	0.162	100	**0.933**	**0.642**	**76**	0.694	0	98	0.770	0.365	99	0.749	0.214	100
COL1A2	0.996	0.583	98	0.843	0.551	99	0.993	0.752	95	0.994	0.180	98	0.955	0.210	100	0.984	0.388	99
FBN1	**0.937**	**0.664**	**99**	0.839	0.347	99	0.834	0.324	97	0.863	0.225	99	0.824	0.600	99	0.890	0.449	99
LDLR	0.791	0	99	0.812	0.374	99	0.745	0.242	95	0.854	0	99	0.841	0.444	99	0.869	0.425	99
RYR1	0.757	0.250	97	0.795	0.364	99	0.885	0.486	80	0.783	0	94	0.856	0.588	99	0.815	0.415	99
SCN5A	0.313	0.242	98	0.301	0.169	99	**0.948**	**0.695**	**76**	0.807	0	95	0.829	0.517	99	0.859	0.285	99
**Mean**	0.776	0.249	97	0.735	0.314	89	0.894	0.575	77	0.833	0.101	94	0.822	0.465	99	0.837	0.392	99

a Len, peptide length parameter of the model; MNA, MNA level parameter of the model; AUC, area under the ROC curve; MCC, Matthew correlation coefficient; %, percentage of the tools’ scores presented in dbNSFP4.1a. The best protein performance for the individual methods are in bold.

We also made a 3D-charts for the PS models performance, where the area of optimal parameter values for a particular protein can be seen (Supplementary Figs S4–S13 in Supplementary Materials), the bumps on the plane reflect the moments when the MNA levels do not capture adjacent a.a. residues, which leads to a decrease in AUC.

### 3.2 Comparison with individual and consensus methods

As mentioned previously, our total dataset contains 3917 unique amino acid substitutions, whose gene annotations were downloaded from dbNSFP4.1a (Liu *et al.* 2020) using search_dbNSFP41a.jar (Table 1). Then, for the canonical isoforms, the prediction scores for individual tools such as SIFT 4G (Vaser et al. 2016), Polyphen2 HDIV (Adzhubei *et al.* 2013), MutationAssessor (Reva *et al.* 2011), PROVEAN (Choi *et al.* 2012) FATHMM (Shihab *et al.* 2013), MVP (Qi *et al.* 2021), LIST-S2 (Malhis *et al.* 2020), MutPred (Pejaver *et al.* 2020), and metapredictors M-CAP (Jagadeesh *et al.* 2016), MetaSVM (Kim *et al.* 2017), MetaLR (Kim *et al.* 2017) were fetched. Although some of the methods lacked annotations for a couple of proteins, the overall dataset coverage was sufficient. The indicated coverage was calculated on the full dataset. For comparison, we used one model for each protein in accordance with the optimal combination of AUC and MCC values in the 5F-CV procedure.

**Figure 5.**
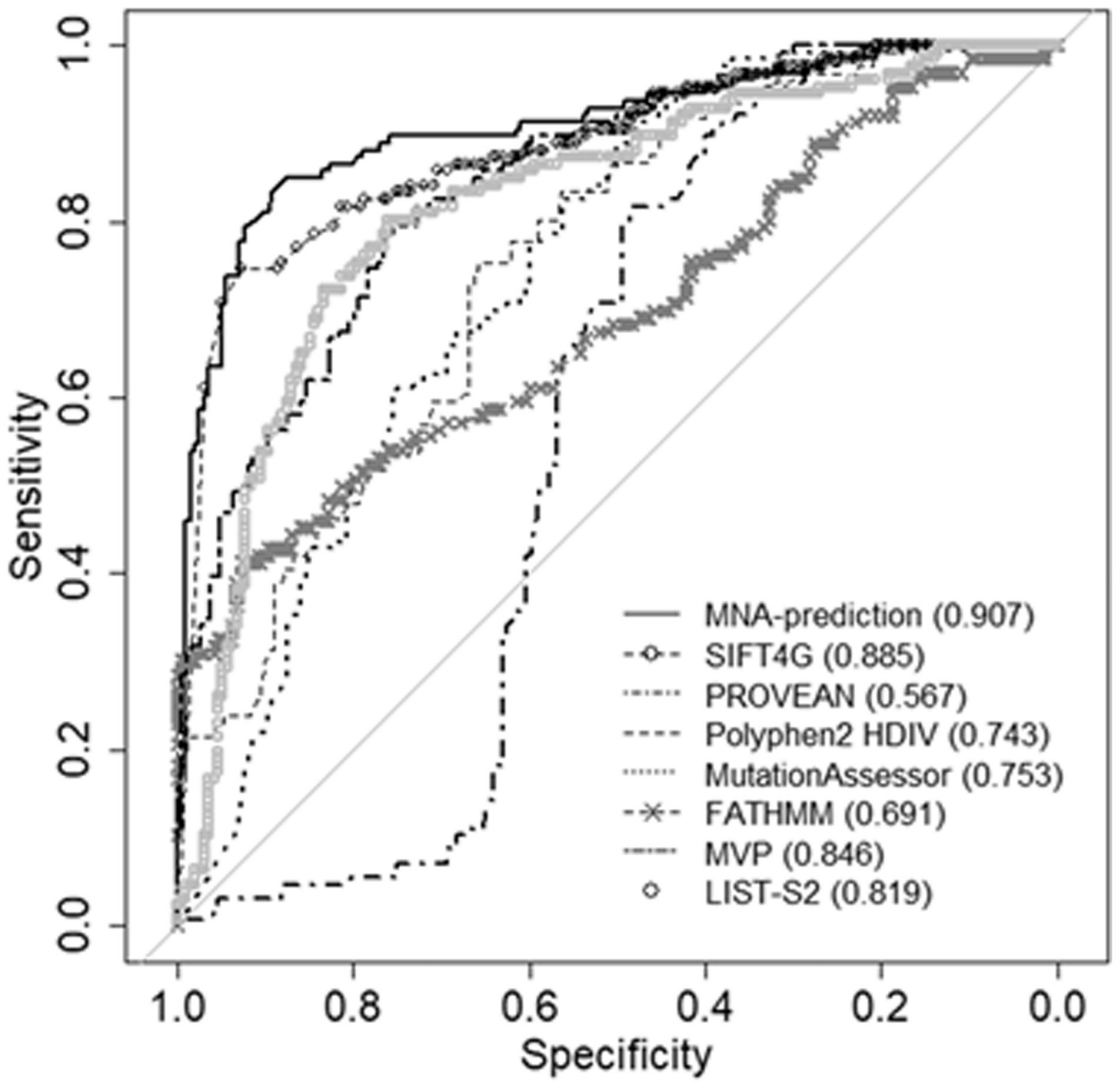
An example comparison of individual methods in predicting the pathogenicity effect of SAVs in P38398 (BRCA1). The area under the receiver operating characteristic curve is in the brackets. MNA-based predictions AUC showed the greatest value.

Generally, the individual predictors demonstrated acceptable results of the classification. MNA-based predictions showed no significant differences in the pairwise MCC comparison with methods other than FATHMM (Student's *t*-test, *P* < 0.05). While SIFT 4G and Polyphen2 got the highest averages, in the protein scope our approach and PROVEAN yielded most of the highest scores (Table 3). MutPred, that predicts the molecular basis of diseases, also achieved a great accuracy, but the poorest coverage may indicate a decreased applicability domain (Table 4). As expected, metapredictors have prevailed mean AUC, in some conditions give a lower prediction power in MCC terms. Since MNA-based scores were obtained in the 5F-CV procedure, and it is not known whether dataset variants were used in the rest predictors trainings, the results of other methods may be overestimated in comparison with the proposed method. Nevertheless, MNA-based models for some proteins received the highest accuracy (Fig. 5, Tables 3 and 4). Remarkably, PROVEAN is also focused on predicting the functional effect of amino acid substitutions using sequence and evolutionary features, and in the case where it failed, the proposed structure-based method worked well. It should be also noted that FATHMM, PROVEAN, MVP, and M-CAP derive inconsistent estimates (MCC ≤ 0), despite the rather high average AUC. Therefore, researchers should choose an individual method carefully and then annotate specific SAVs.

We observed the cases in which the MNA-based method gave the correct prediction results, while other methods failed. Some of these variants are given in Table 5. To explain the results, we determined the localization of these substitutions in the proteins and examined the features of their environment using UniProt feature viewer (Supplementary Fig. S14). It turned out that all the substitutions were located in specific areas, such as the functional region (Supplementary Fig. S14A), the disulfide bond (Supplementary Fig. S14B) or the helix (Supplementary Fig. S14C). Notably, pathogenic SAVs in BRCA2 and LDLR are mapped within unique peptides, whereas the benign SAV in CFTR is outside. In the first case isoleucine replaces methionine and both these residues belong to the nonpolar (hydrophobic) a.a. group, therefore such a substitution is considered conservative. The same picture is observed for Arg to Lys (both residues belong to the group of positively charged amino acids) and Ile to Phe [both residues belong to the group of nonpolar (hydrophobic) amino acids] substitutions. It is likely that our approach better captures changes in the structural properties of such areas.

**Table 5.** Examples of correctly predicted variants that were incorrectly assessed by the other 11 methods.^a^

Gene	Substitution	Score	*N*	Clinical sign.	rs dbSNP
BRCA2	Met1168Ile	0.263	0	Likely pathogenic	rs1555283267
LDLR	Arg88Lys	0.680	4^b^	Pathogenic	rs1398808477
CFTR	Ile285Phe	–0.627	0	Benign	rs151073129

a Score, MNA-based prediction; *N*, methods correctly predicted pathogenicity.

b FATHMM, MVP, M-CAP (all LDLR variants considered as pathogenic), and MetaLR.

## 4 Discussion 

In this study, we have examined the feasibility of utilizing the structure-activity paradigm to predict the pathogenic effect of SAVs. For this purpose, 10 proteins associated with various pathological conditions in humans were selected. The choice was made based on the total number of the clinically annotated variants (minimum 125 SAVs) along with the availability of comparative assessment provided by other methods. In cheminformatics, even 10 structural formulas of molecules may be enough to create reasonable SAR models. The classification models were trained with variable parameters of the MNA descriptors levels and the peptide frame of the substituted a.a. residues and their surroundings; the search for the optimal parameters was carried out.

Finally, the 5-fold CV prediction results of the best models were compared with eleven computational prediction algorithms: eight individual [SIFT 4G (Vaser et al. 2016), Polyphen2 HDIV (Adzhubei *et al.* 2013), MutationAssessor (Reva *et al.* 2011), PROVEAN (Choi *et al.* 2012), FATHMM (Shihab *et al.* 2013), MVP (Qi *et al.* 2021), LIST-S2 (Malhis *et al.* 2020), MutPred (Pejaver *et al.* 2020)] and three ensemble [M-CAP (Jagadeesh *et al.* 2016), MetaSVM (Kim *et al.* 2017), MetaLR (Kim *et al.* 2017)]. The MNA-based approach showed the similar predictive accuracy with the best of them in terms of AUC and MCC, albeit for a limited set of subjects under study. MNA descriptors have proven capable of fully describing large linear molecules such as peptides. Practically, by utilizing the properties of the primary structure alone, it was possible to achieve a comparable accuracy with other individual methods using the properties of amino acid sequences (Adzhubei *et al.* 2013, Qi *et al.* 2021). This can be explained by the relations of the secondary, tertiary, and primary structures properties. The obtained values are in agreement with the PS predictors of other authors (Crockett *et al.* 2012, Riera *et al.* 2016), although a direct comparison could not be provided. According to 5F-CV, LOO-CV, 20F-CV results and dataset size versus accuracy (Supplementary Table S1, Supplementary Fig. S2C), it can be concluded that MNA-based performance does not correlate with the number of pathogenic variants in the training set. This can be seen as an advantage of the SAR approach as for many proteins there are only a few clinically annotated SAVs. The described results were achieved without the direct use of information about 2D or 3D structures of proteins, as well as the alignment or search for homologues. As already mentioned, we tested the capabilities of the SAR approach, so we deliberately did not use any evolutionary information, which is reported by many studies to make a significant contribution to predictions of pathogenic effect (Capriotti *et al.* 2013, Niroula *et al.* 2015). In perspective, the MNA-based approach may be improved by suppling additional features such as the evolutionary conservation, data on regulatory and binding sites, 2D and 3D structures of proteins.

## Supplementary Material

btad484_Supplementary_Data

## Data Availability

ClinVar https://www.ncbi.nlm.nih.gov/clinvar/. humsavar https://www.uniprot.org/docs/humsavar. dbNSFP v.4.1a http://database.liulab.science/dbNSFP.
