# Supplementary material for: Prediction of pathogenic single amino acid substitutions using molecular fragment descriptors
Source: Bioinformatics. 2023 Aug 3;39(8):btad484. doi: 10.1093/bioinformatics/btad484 (PMC10435372; doi:10.1093/bioinformatics/btad484)
Supplement: btad484_Supplementary_Data [file btad484_supplementary_data.zip › Sup_Matirials.pdf]

Supplement

to the paper of Anton Zadorozhnyy, Anton Smirnov, Dmitry Filimonov and Alexey Lagunin
"Prediction of pathogenic single amino acid substitutions using molecular fragment descriptors"

Contents

1. **Table 1S.** The accuracies of self-recognition test, the unmutated peptide tests and the cross-validation procedures. 2
2. **Figure 1S.** Performance in the context of peptide length (A) and MNA level (B) parameters 3
3. **Figure 2S.** Comparison of methods in predicting the effect of AA substitutions (colored) 4
4. **Figure 3S.** MNA-based prediction accuracy relative to protein primary structure 5
5. **Figure 4S.** Prediction amino acid substitutions in ATM 6
6. **Figure 5S.** Prediction amino acid substitutions in ATP7B 7
7. **Figure 6S.** Prediction amino acid substitutions in BRCA1 8
8. **Figure 7S.** Prediction amino acid substitutions in BRCA2 9
9. **Figure 8S.** Prediction amino acid substitutions in RYR1 11
10. **Figure 9S.** Prediction amino acid substitutions in COL1A1 12
11. **Figure 10S.** Prediction amino acid substitutions in SCN5A 13
12. **Figure 11S.** Prediction amino acid substitutions in FBN1 14
13. **Figure 12S.** Prediction amino acid substitutions in CFTR 15
14. **Figure 13S.** Prediction amino acid substitutions in LDLR 16
15. **Figure 14S.** Uniprot feature viewer zoomed in positions from Table 4 17
16. **Figure 15S.** Colored example comparison of the individual methods in predicting the pathogenicity effect of SAVs in P38398 (BRCA1). 18

Table 1S. The accuracies of self-recognition test, the unmutated peptide tests and the cross-validation procedures.

Gene	Sen.	Spec.	BA	TN_{REF}	FP_{REF}	$Spec._{REF}$	$Spec._{5F-CV}$	LOO-CV	20F-CV	5F-CV [min;max]
ATM	1.000	1.000	1.000	189	86	0.687	0.547	0.631	0.641	0.627 [0.503;0.726]
ATP7B	0.991	1.000	0.995	199	76	0.724	0.765	0.815	0.808	0.807 [0.753;0.909]
BRCA1	1.000	0.99	0.995	263	12	0.956	0.923	0.900	0.894	0.907 [0.836;0.929]
BRCA2	1.000	0.988	0.994	163	112	0.593	0.728	0.780	0.775	0.795 [0.710;0.805]
CFTR	0.967	1.000	0.984	140	135	0.509	0.633	0.712	0.719	0.690 [0.535;0.714]
COL1A2	1.000	1.000	1.000	218	57	0.793	1.000	0.992	0.993	0.992 [0.972;1.000]
FBN1	0.997	1.000	0.999	234	41	0.851	0.724	0.789	0.787	0.795 [0.693;0.885]
LDLR	0.973	0.988	0.981	219	56	0.796	0.709	0.730	0.711	0.732 [0.699; 0.779]
RYR1	0.996	1.000	0.998	193	82	0.702	0.830	0.875	0.870	0.871 [0.807;0.909]
SCN5A	0.991	1.000	0.996	187	83	0.693	0.703	0.724	0.728	0.765 [0.666;0.837]
Mean	0.992	0.997	0.994	201	74	0.730	0.756	0.795	0.793	0.798 [0.717;0.849]

Metrics of self-recognition test: Sen. – Sensitivity; Spec. – Specificity; BA – Balanced accuracy.

Metrics of the reference peptides test: TN_{REF} – True negative; FP_{REF} – False positive; $Spec._{REF}$ – Specificity. The unmutated peptides were randomly extracted from the reference protein sequences.

Metrics of the cross-validation procedures: LOO CV – leave-one-out cross-validation; 20F-CV – five-fold cross-validation; 5F-CV – five-fold cross-validation, mean AUC values with range AUC values in the brackets.

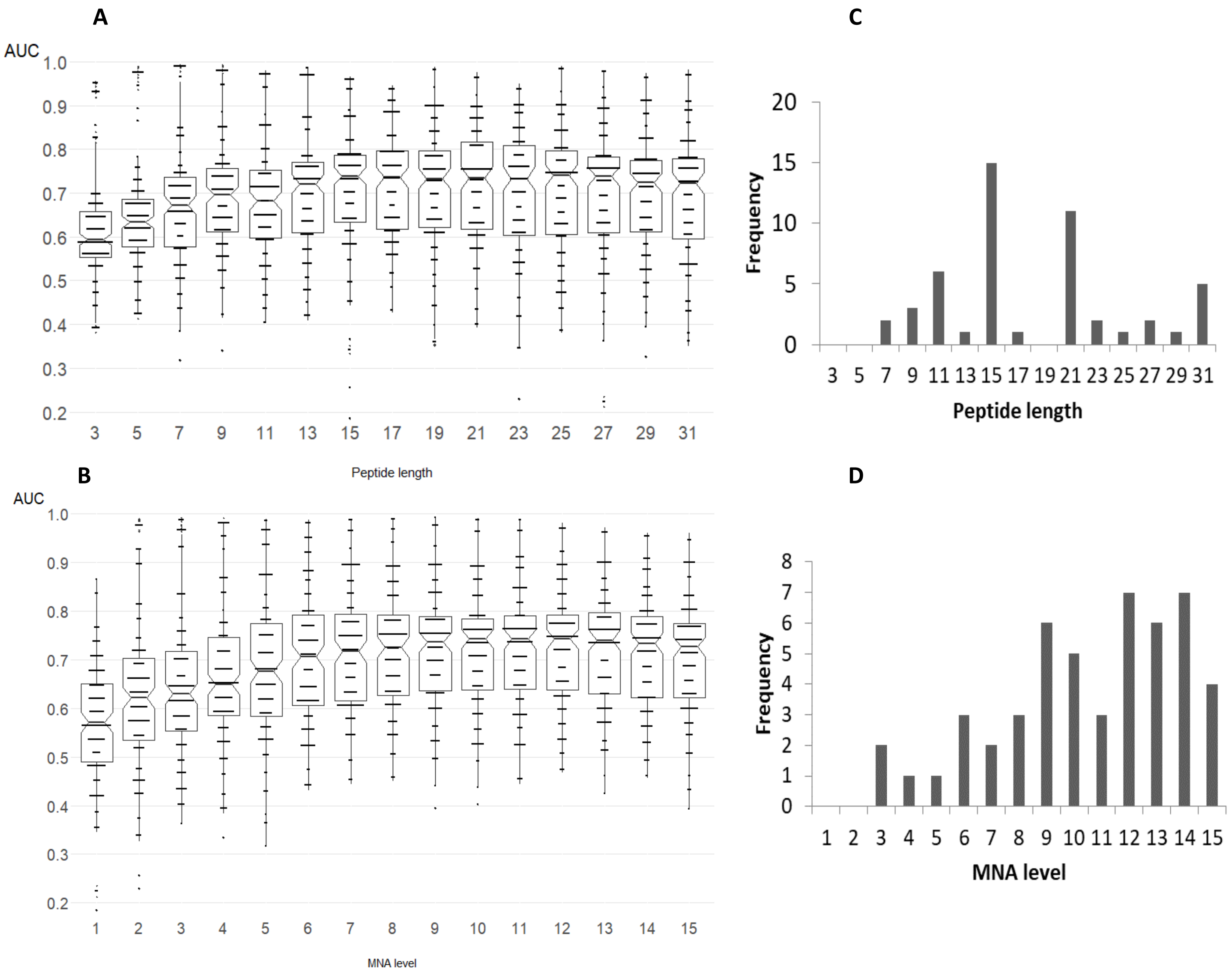

Figure 1S. A, B – Joint five-fold cross-validation performance in the context of peptide length (A) and MNA level (B) parameters. Observed similar median value of AUC, as well as the first and third quartiles, with the exception of 1-4 MNA levels and the shortest lengths of peptides.

C, D – Frequency of occurrence MNA and length parameters in fifty best models (five per protein).

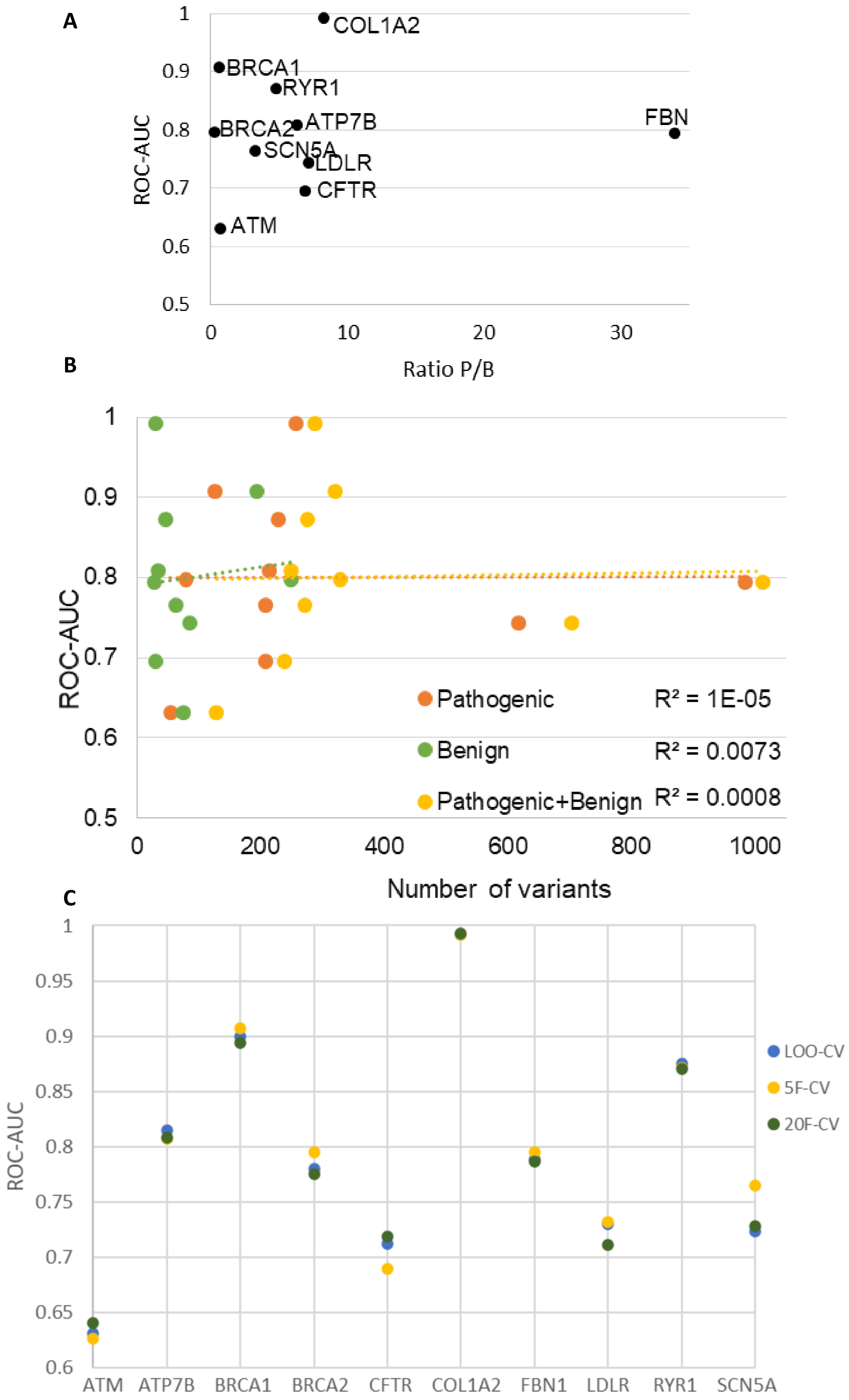

Figure 2S. Dependency of AUC values of the best protein-specific SAR models on: (A) – The ratio of pathogenic/benign variants in the training sets; (B) – The number of benign/pathogenic variants in the training sets; (C) – Cross-validation procedures. LOO CV – leave-one-out cross-validation; 5F-CV – five-fold cross-validation; 20F-CV – twenty-fold cross-validation.

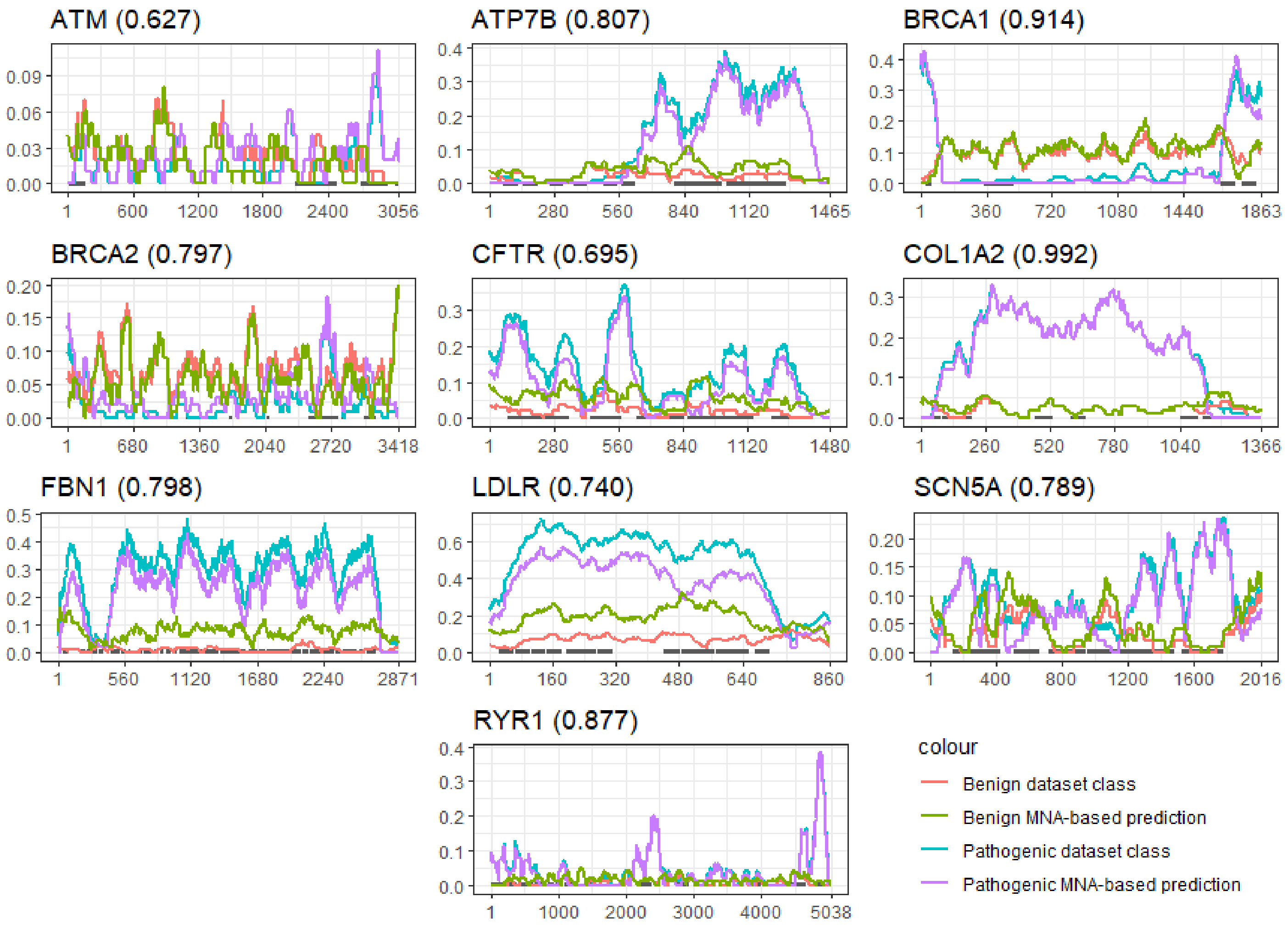

Figure 3S. MNA-based prediction accuracy relative to protein primary structure. Axis X ─ protein length in amino acids with known domains localization as black lines, axis Y ─ percentage of known amino acid substitutions with the appropriate annotation in 100 a.a. sliding window. AUC values of the best MNA-based model at 5F-CV are represented in the brackets.

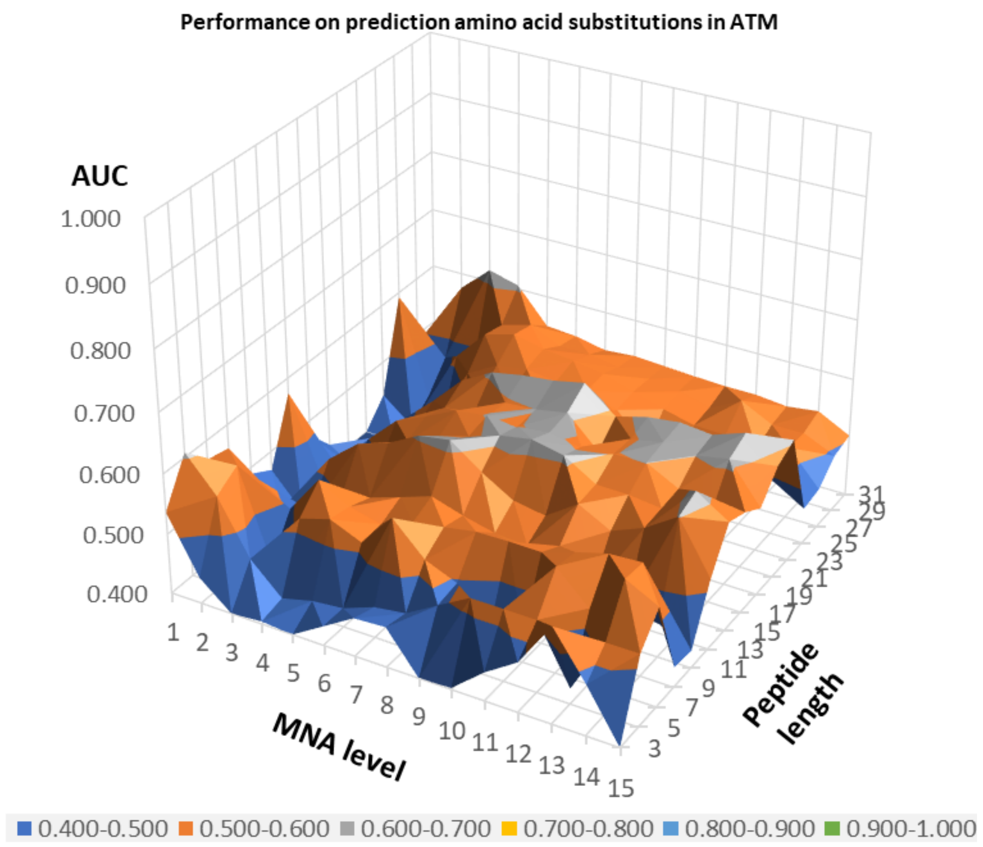

Figure 4S. Method performance on prediction amino acid substitutions in ATM

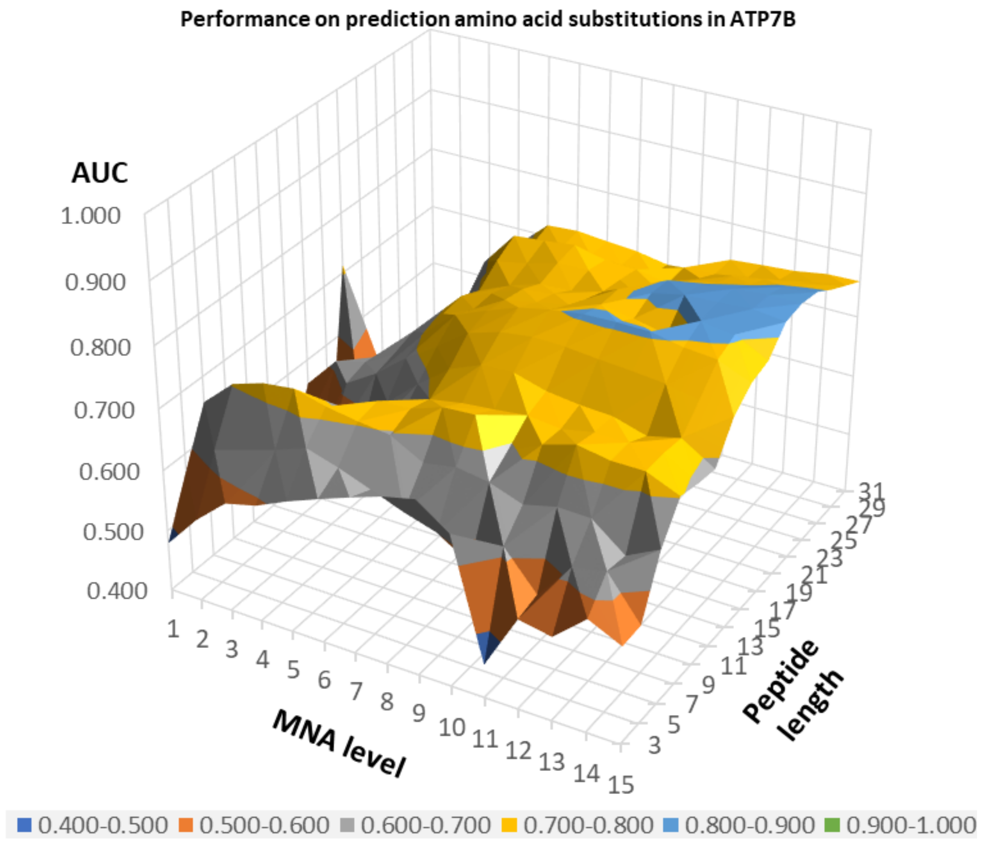

Figure 5S Method performance on prediction amino acid substitutions in ATP7B

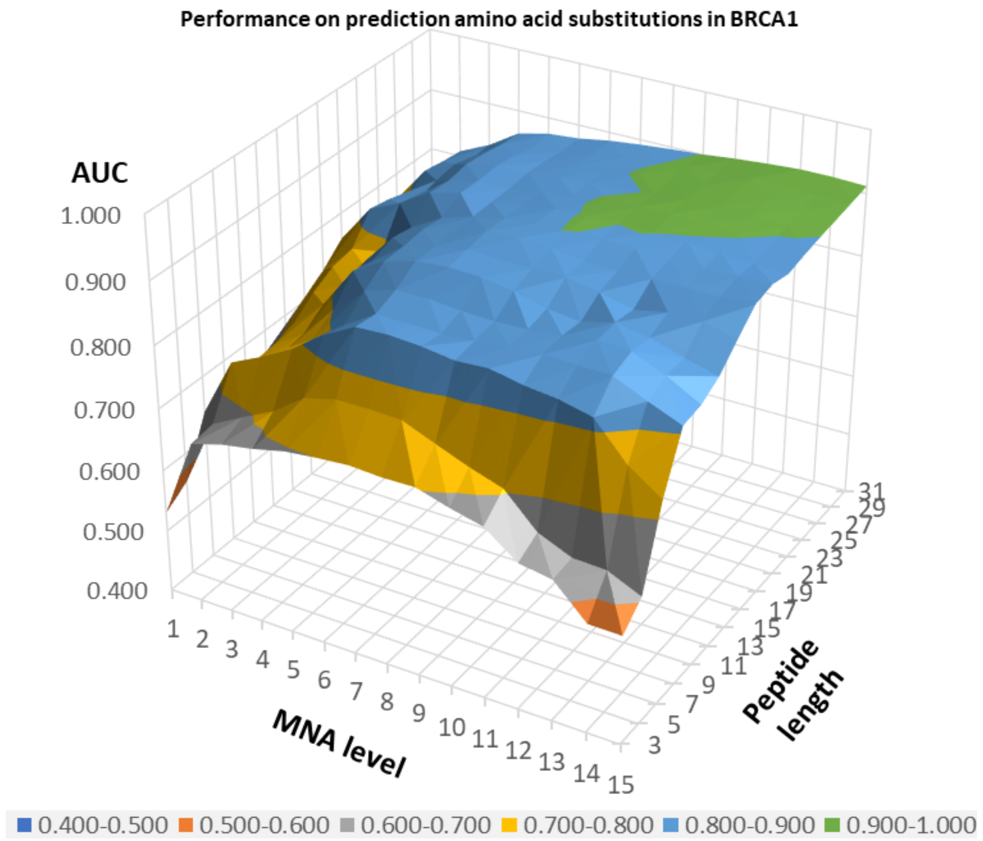

Figure 6S. Method performance on prediction amino acid substitutions in BRCA1

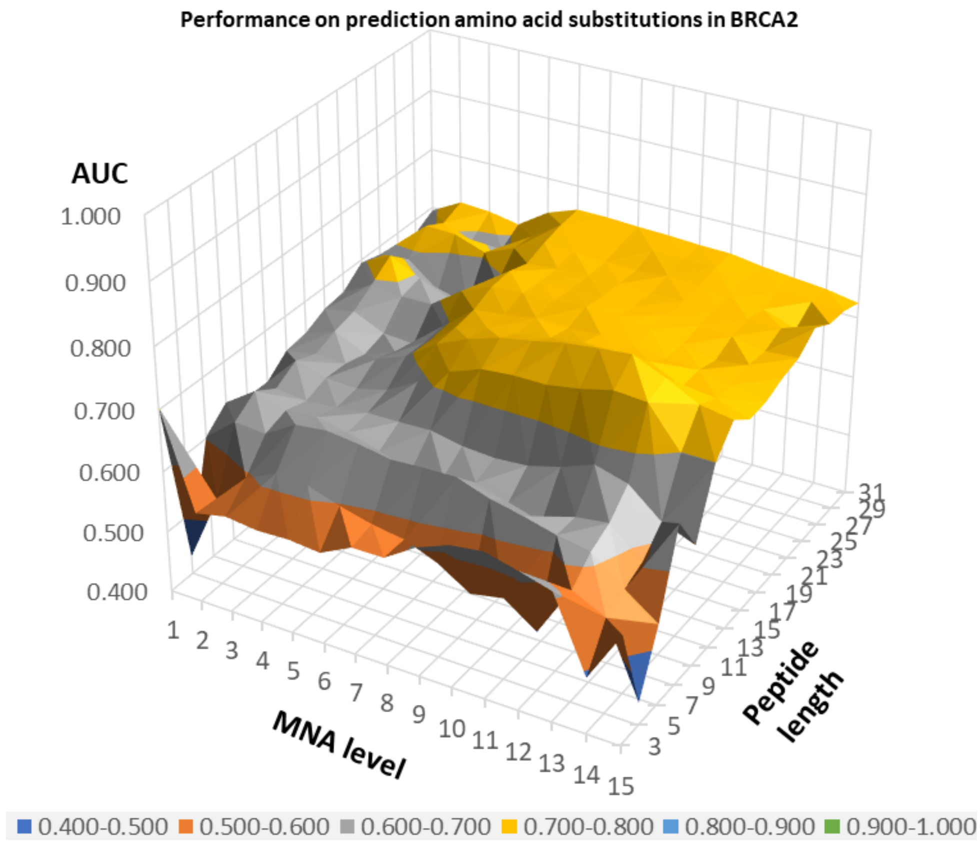

Figure 7S. Method performance on prediction amino acid substitutions in BRCA2

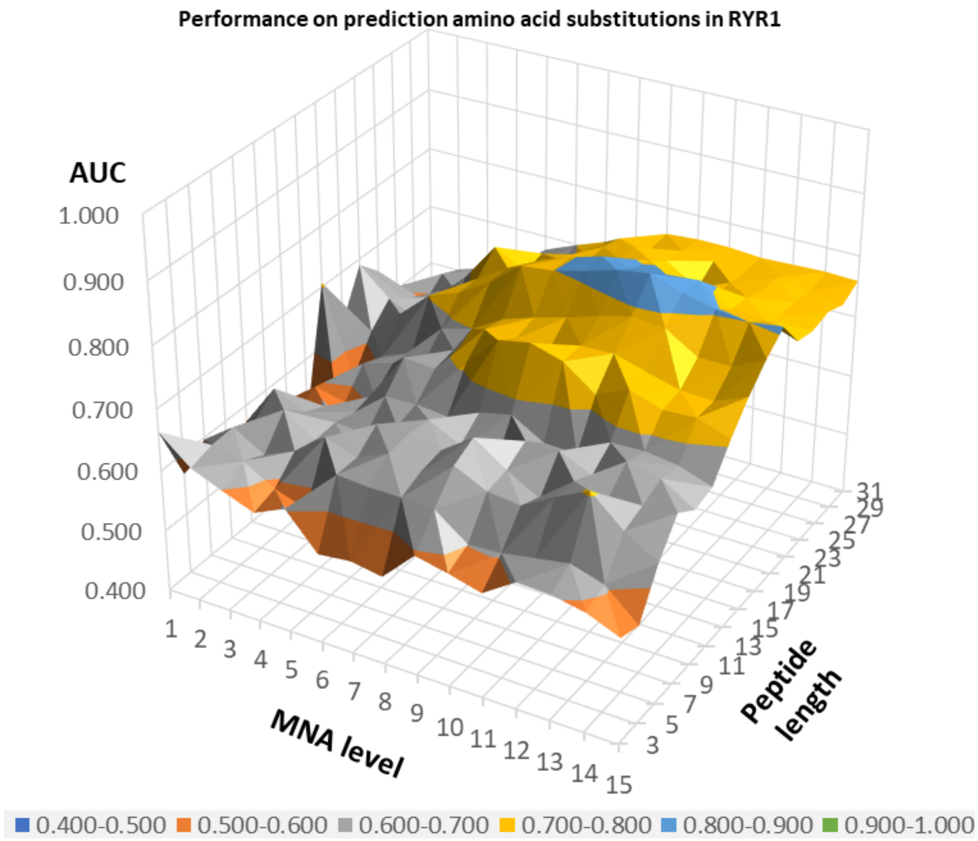

Figure 8S Method performance on prediction amino acid substitutions in RYR1

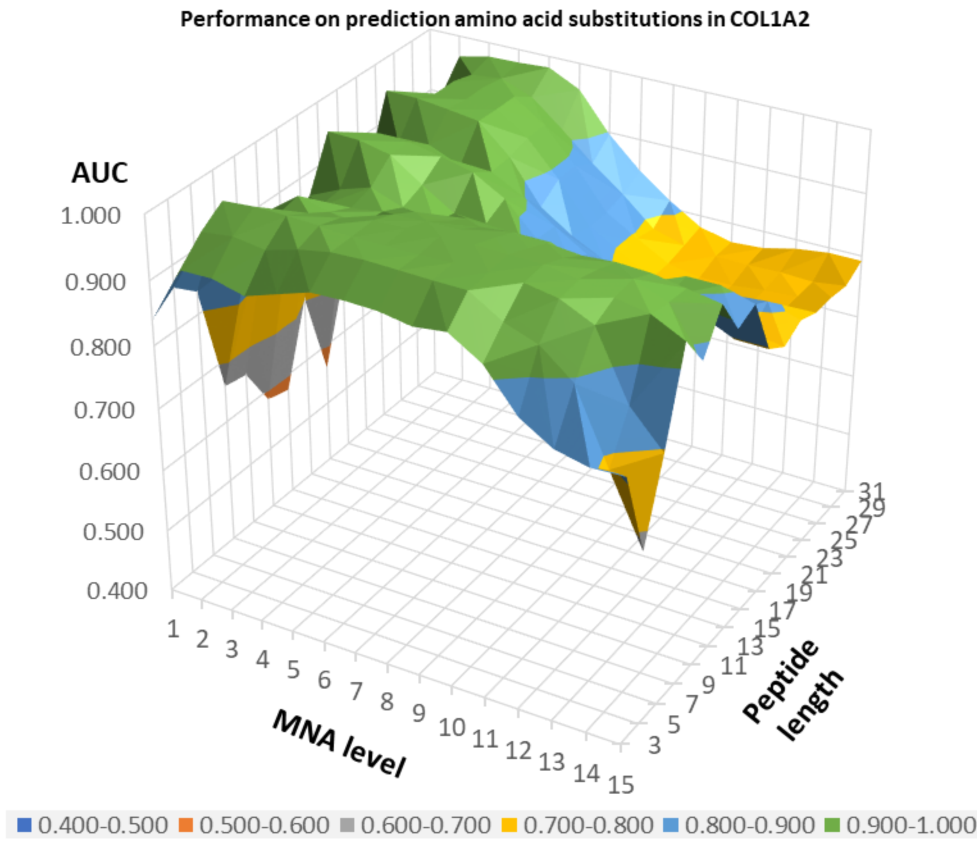

Figure 9S. Method performance on prediction amino acid substitutions in COL1A2**.**

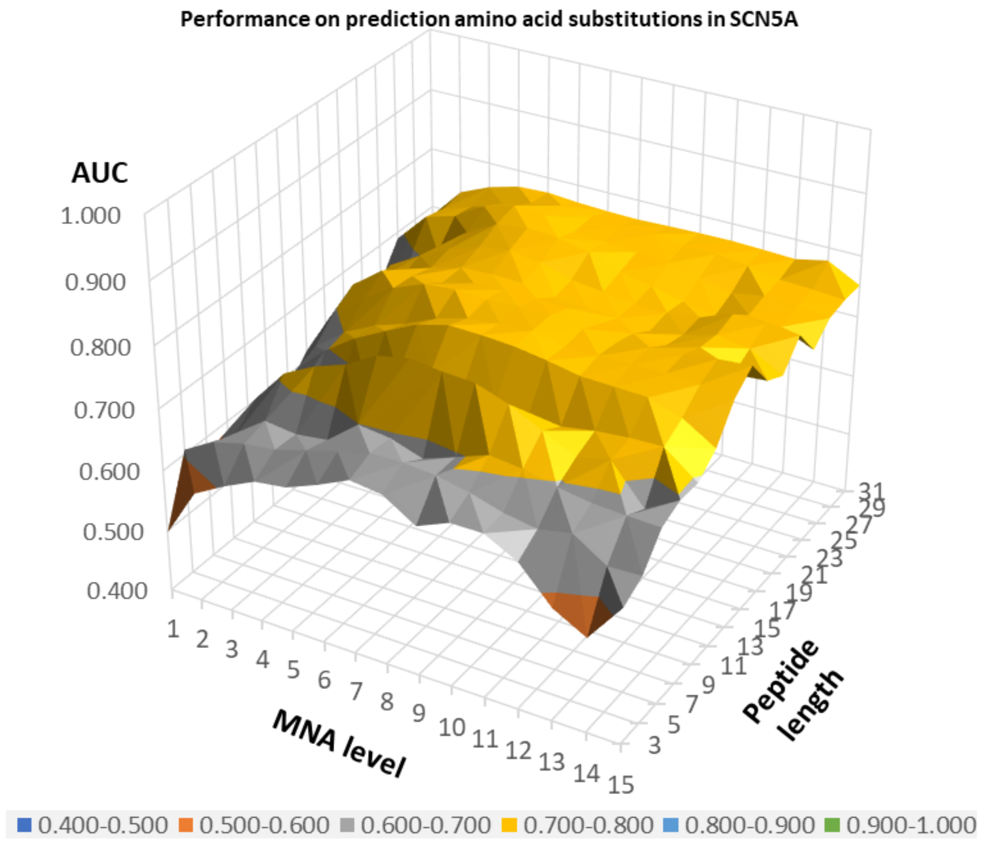

Figure 10S. Method performance on prediction amino acid substitutions in SCN5A

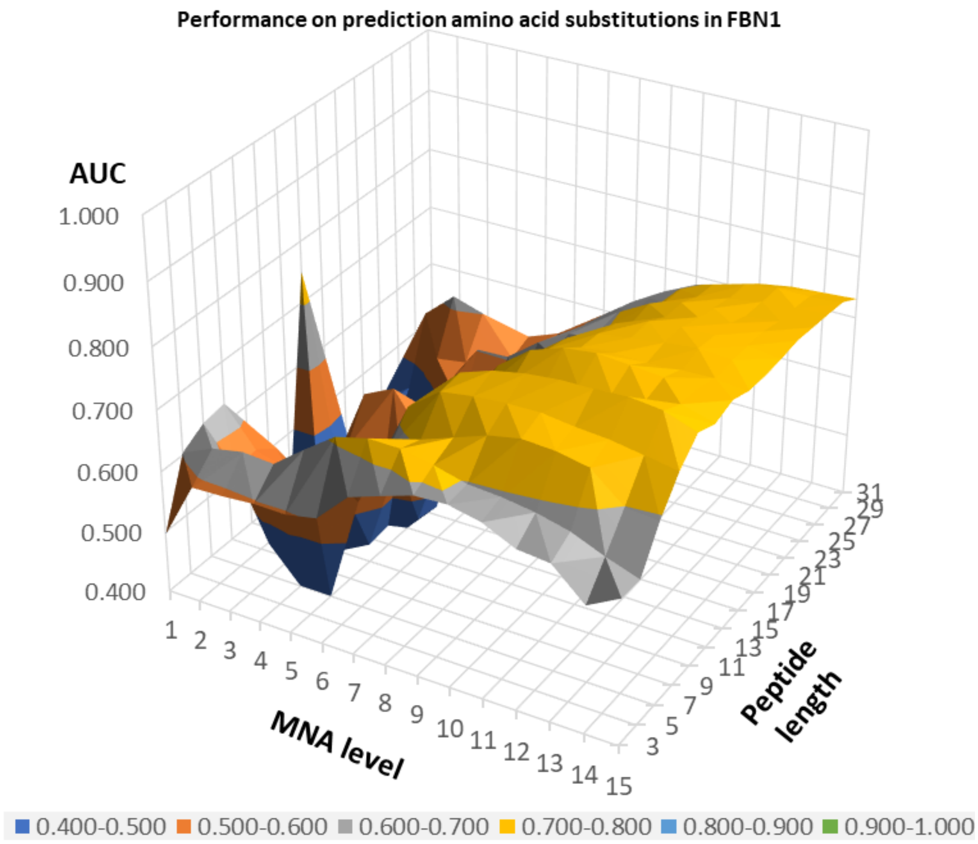

Figure 11S. Method performance on prediction amino acid substitutions in FBN1

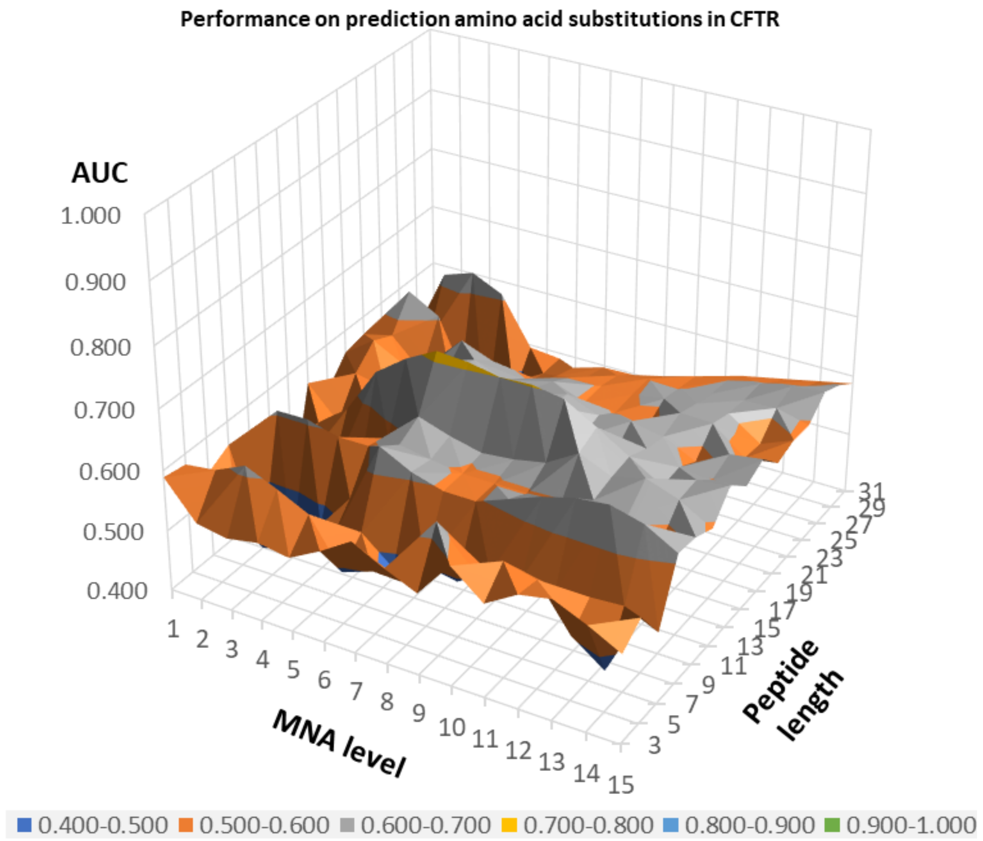

Figure 12S. Method performance on prediction amino acid substitutions in CFTR

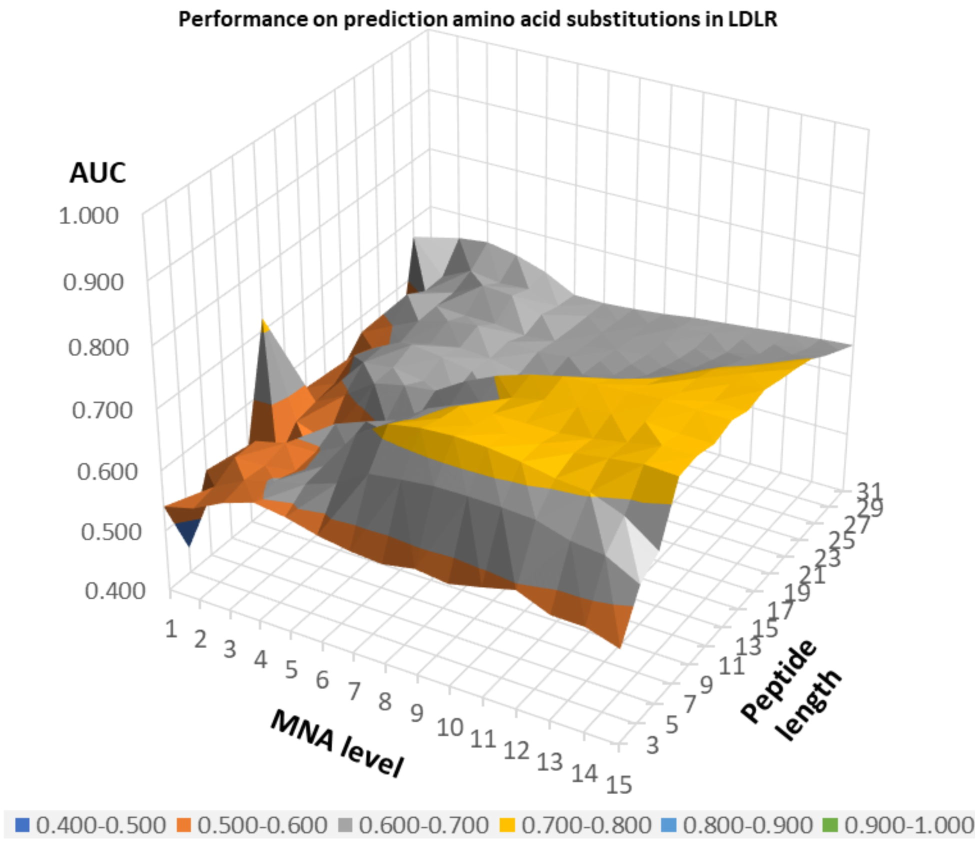

Figure 13S. Method performance on prediction amino acid substitutions in LDLR

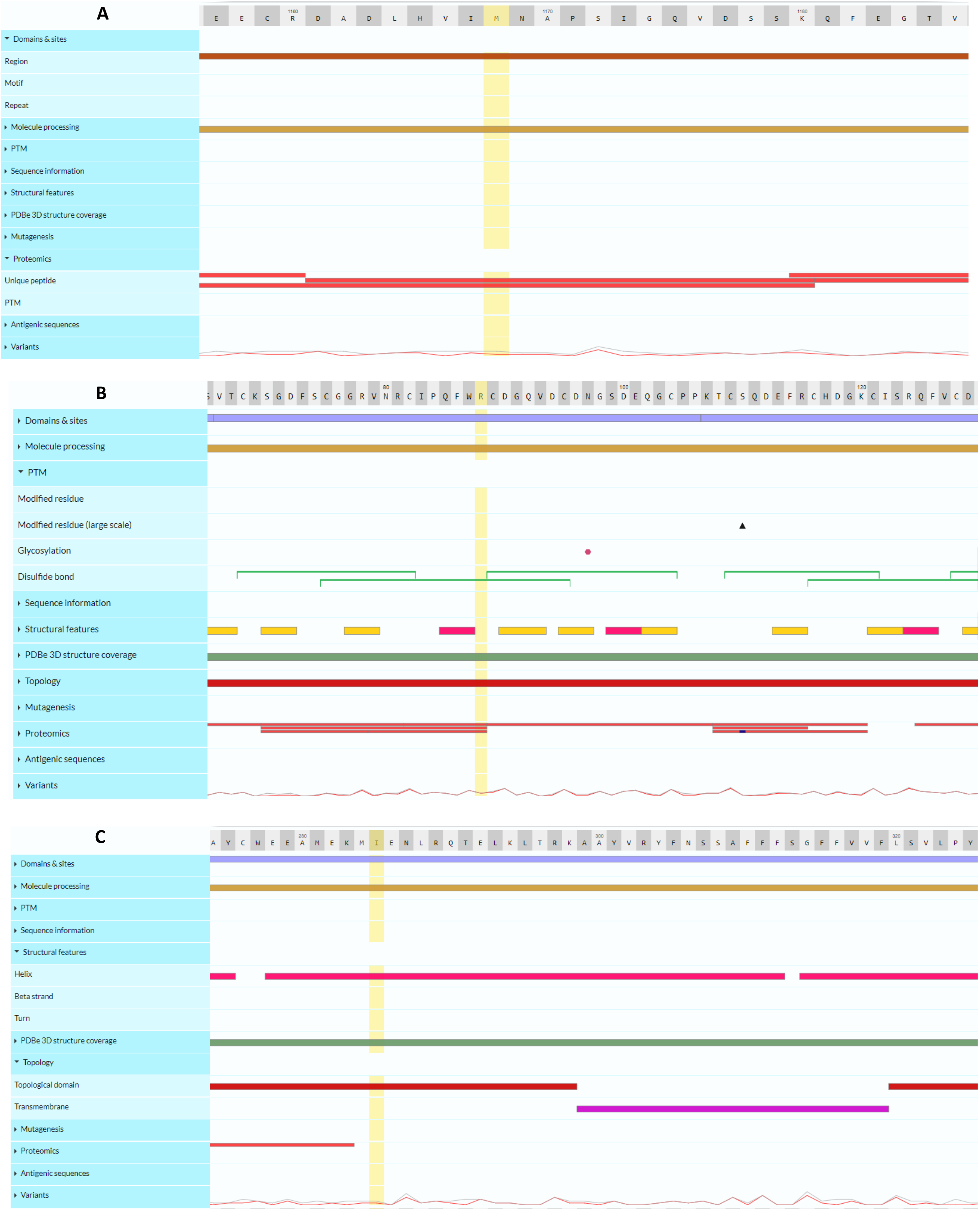

Figure 14S UniProt feature viewer zoomed in positions from Table 4: A – BRCA2, Met1168Ile; B – LDLR, Arg88Lys; C – CFTR, Ile285Phe.

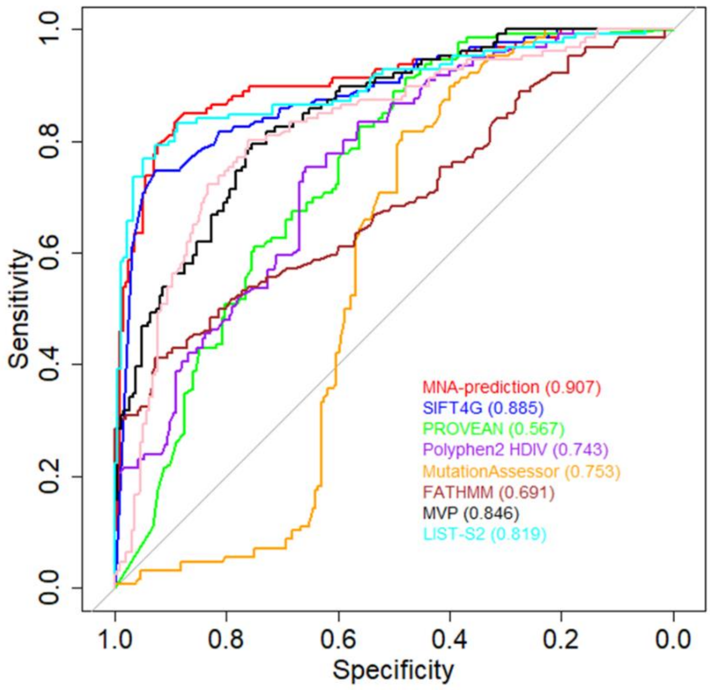

Figure 15S. Comparison of methods in predicting the effect of AA substitutions in P38398 (BRCA1). Area under the receiver operating characteristic curve in the brackets.
